# Impedance biosensor for *real-time* monitoring and prediction of thrombotic individual profile in flowing blood

**DOI:** 10.1371/journal.pone.0184941

**Published:** 2017-09-18

**Authors:** Denise De Zanet, Monica Battiston, Elisabetta Lombardi, Ruben Specogna, Francesco Trevisan, Luigi De Marco, Antonio Affanni, Mario Mazzucato

**Affiliations:** 1 Polytechnic Department of Engineering and Architecture, University of Udine, Udine, Italy; 2 Department of Translational Research, Stem Cells Unit, National Cancer Institute CRO - IRCCS, Aviano, Pordenone, Italy; Queen’s University at Kingston, CANADA

## Abstract

A new biosensor for the *real-time* analysis of thrombus formation is reported. The fast and accurate monitoring of the individual thrombotic risk represents a challenge in cardiovascular diagnostics and in treatment of hemostatic diseases. Thrombus volume, as representative index of the related thrombotic status, is usually estimated with confocal microscope at the end of each *in vitro* experiment, without providing a useful behavioral information of the biological sample such as platelets adhesion and aggregation in flowing blood. Our device has been developed to work either independently or integrated with the microscopy system; thus, images of the fluorescently labeled platelets are acquired in *real-time* during the whole blood perfusion, while the global electrical impedance of the blood sample is simultaneously monitored between a pair of specifically designed gold microelectrodes. Fusing optical and electrical data with a novel technique, the dynamic of thrombus formation events in flowing blood can be reconstructed in *real-time*, allowing an accurate extrapolation of the three-dimensional shape and the spatial distribution of platelet thrombi forming and growing within artificial capillaries. This biosensor is accurate and it has been used to discriminate different hemostatic conditions and to identify weakening and detaching platelet aggregates. The results obtained appear compatible with those quantified with the traditional optical method. With advantages in terms of small size, user-friendliness and promptness of response, it is a promising device for the fast and automatic individual health monitoring at the Point of Care (POC).

## Introduction

Blood coagulation is a complex mechanism whose alteration can produce diseases such as thrombosis, embolisms and hemorrhages. In particular, thrombosis is one of the major causes of death and implicates a series of morphological and functional changes occurring in different temporal and spatial dimensional scales [1]. Platelets play a central hemostatic role, since, once activated, they rapidly adhere to a damaged substrate and aggregate forming thrombi [2, 3].

The advances in technologies and computational methods have moved the cell biology toward a quantitative view [4, 5]: there is an increasing interest for an *in vitro* measurement of coagulation process [6, 7] in order to determine, as quickly as possible, the risk of thrombosis or bleeding, expecially during a surgical treatment. On the other hand, moving the attention toward diagnostics, it is becoming essential to evaluate thrombus formation under flow conditions [8–14] to study the individual thrombotic process in humans, with the goal of development of innovative screening platforms for personalized pharmacological treatments. The improvements in microscope technologies and computational techniques now make possible to investigate platelets functionality and thrombus formation [4, 15]. If the specific aim is to measure the volumetric structure of an object, confocal microscope provides as output a sequence of fluorescent images, called *z*-stack, acquired along the vertical *z*-dimension, that should facilitate the three-dimensional (3D) volume reconstruction and measurement, rejecting the out-of-focus fluorescence light. The accuracy and the standardization of thrombus volume measurements, starting from confocal microscope images, have always been open and problematic issues in the specific biological field of blood coagulation. However, the image acquisition and post process from confocal microscope is a costly and laborious methodology; for this reason its application in clinics is restricted.

In recent years, medical research has gradually oriented its attention to new non-invasive and *real-time* devices able to assess the thrombotic risk profile by reconstructing the dynamic of thrombus formation events. A preliminary electrical characterization of thrombus formation in microchannels under blood flow conditions using electrical impedance spectroscopy [16] has been presented in [17] by using an impedance meter and a low cost sensor based on copper printed circuit board technology and recently improved, as reported in [18–23].

This paper further explores the relevance of blood impedance technique in measuring thrombus formation under flow conditions. Here a new biosensor is presented, cheaper than the confocal microscope, capable to characterize, with a non-invasive and *real-time* impedance analysis, the dynamic of platelet adhesion, aggregation and thrombus formation. A novel, fast and adaptive method is described to quantify volumes from confocal microscope images with accuracy and standardization, thus providing a point of reference for the new device calibration and validation. From impedance measurements, it is possible to reconstruct the 3D spatial and temporal distribution of thrombi using a specifically developed analysis tool, named FUSEIT, based on a finite-elements-like method [19, 22, 23]. Volume measurements obtained with FUSEIT highly match volumes reconstructed from confocal microscope images acquisitions. In addition, our device is capable of discriminating different hemostatic conditions and of identifying weakening and detaching aggregates, as demonstrated by preliminary results here presented.

## Materials and methods

### Blood sample preparation

For each experiment, 2 ml of venous blood are collected into D-Phenylalanyl-L-Prolyl-L-Arginine Chloromethyl Ketone dihydrochloride (PPACK, produced by Calbiochem, La Jolla, CA, 50 *μ*mol final concentration). Informed written consent have been obtained from healthy blood donors according to the Declaration of Helsinki and the DMS of the Italian Ministry of Health, November 2*^nd^*, 2015, (quality and safety about blood and blood donors). Moreover, the Ethics Committee CRO-IRCCS Aviano, Code Number CRO-2014-56, approved all the studies using human blood samples and, therefore, this one. Afterwards, 2 *μ*l drop of acid-insoluble fibrillar type I collagen (produced by Sigma Aldrich, St. Louis, MO, 1 mg ml^−1^ concentration) is applied as coating substrate for 60 minutes, to induce the aggregation process. The fluorescent dye quinacrine dihydrochloride (mepacrine, Sigma Aldrich, 10 *μ*mol final concentration) is added to whole blood to label platelets.

### Biosensor description

**Fig 1:**
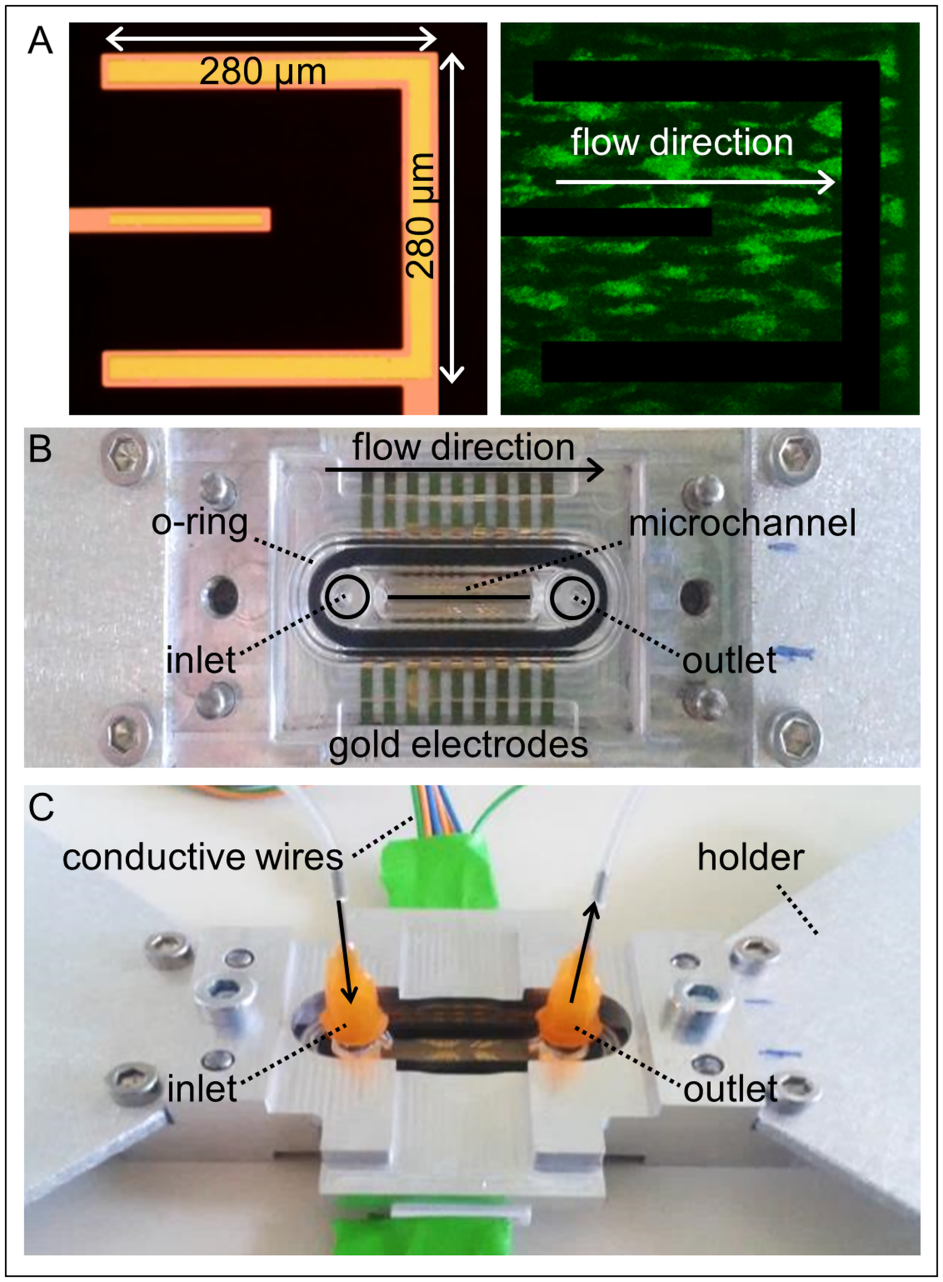
The new biosensor. A: Detail of u-shaped electrodes arrangement (yellow: gold layer, pink: passivation layer) and example of an image acquired with confocal microscope. B: The core of the device consists of a bottom glass with gold electrodes and a polycarbonate chamber containing an o-ring that prevents blood leakages. The bottom glass and the chamber define the microchannel (cross section: 500 x 100 *μ*m^2^) where the blood flows, from the inlet to the outlet. C: Assembled device: an holder for the allocation on the microscope and a component with conductive wires are locked one another holding the glass with electrodes and the polycarbonate chamber inside.

The new biosensor is shown in Fig 1. It allows the simultaneous acquisition of optical images and related impedance data during blood perfusion in an artificial microchannel specifically designed and representing the core of the device. The bottom wall is realized with a glass where u-shaped gold electrodes are sputtered (Fig 1A) and delimit an investigation area of 280 x 280 *μ*m^2^ (outer electrode: 30 *μ*m wide, inner electrode: 20 *μ*m wide). A SiO_2_ passivation layer (pink in Fig 1A) is set over the tracks to delimit the active area of the electrodes. The other walls of the microchannel and the microfluidic inlet and outlet connections are molded in a polycarbonate chamber that contains an o-ring for the fluid leakages prevention towards the contacts (Fig 1B). The blood flows from the inlet to the outlet through a cross section of 500 x 100 *μ*m^2^. A metallic holder and a component with conductive sensing wires are locked one another to complete the assembling of the device (Fig 1C).

### Acquisition system

**Fig 2:**
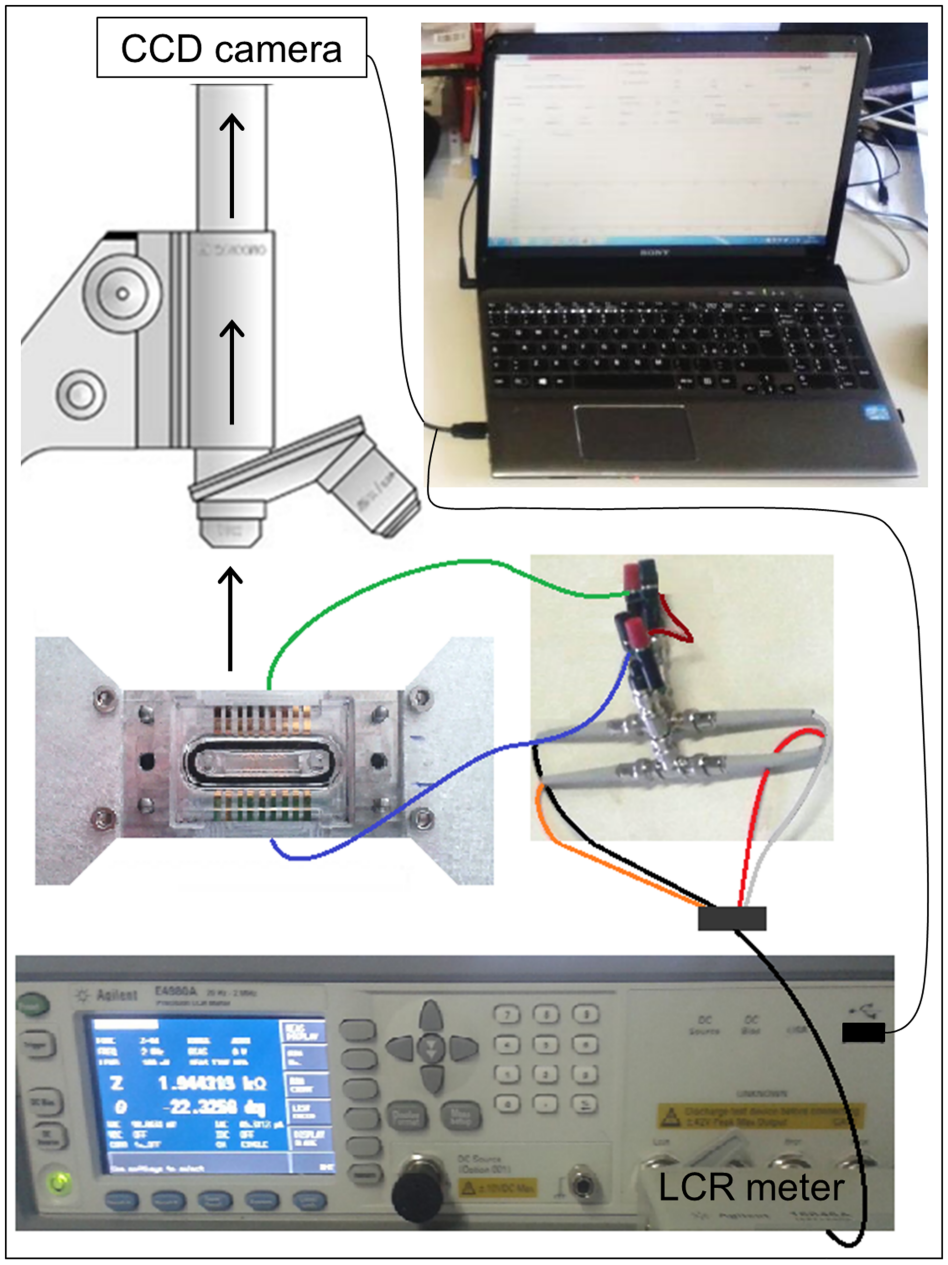
Measurement bench. Optical images are obtained in *real-time* with the CCD camera of the microscope, while the LCR meter acquires impedance values. Optical and electrical information are both connected to a laptop.

The device, connected to a high precision LCR meter [24], is positioned on the stage of a Confocal Laser Scanning Microscope (CLSM, Eclipse TE300, manufactured by Nikon, Tokyo, J) based on Nipkow disk technology (Fig 2). A 40X oil-immersion objective (numerical aperture: 1.30, Nikon) is mounted on a piezoelectric driver controlled by the Andor IQ acquisition software (made by Andor^*TM*^ Technology, Belfast, UK). The physiological conditions typical of the microcirculation districts (arterioles) are reproduced *in vitro* with a controlled fluid perfusion system. In particular, a syringe pump (manufactured by Harvard Apparatus, Boston, MA) aspirates blood from the chamber outlet for a time interval of 300 seconds, at a controlled temperature of 37°C, with a constant flow rate *Q* = 75 *μ*l/min, in order to achieve the physiological shear rate

$$\begin{array}{c} \gamma =\frac{6Q}{wh^{2}}=1500\;s^{-1}, \end{array}$$ (1)

where *w* and *h* are the width (500 *μ*m) and the height (100 *μ*m) of the channel section, respectively.

Optical and electrical data are acquired simultaneously during the blood perfusion through the microchannel. Fluorescent images are digitized in *real-time* with the CCD camera (iXon^*EM*+^, Andor^*TM*^ Technology) of the confocal microscope, whose internal sensor of 512 x 512 pixel detects the intensity emission of the fluorescent dye quinacrine that labels platelets, in response of an excitation laser wavelength of 488 nm. In particular, each pixel in the digital images thus acquired carries an intensity information, represented by an integer in the interval [0, 255]. Regardless of the representation pseudo color (green in this case), such images are composed exclusively of shades of gray, varying from black (0, the weakest intensity) to white (255, the strongest intensity). An appropriate laser power is set in order to achieve an adequate emission intensity, avoiding the pixel saturation in the CCD sensor. Taking into account the magnification determined by the optical path (1 pixel = 0.33 *μ*m), the investigation area results to measure 28547.48 *μ*m^2^. Images are collected during the dynamic experiment every second, for a total time of 300 seconds. The plane of acquisition in the confocal microscope, named focal plane, is identified as the plane where platelets adhere and the electrodes can be seen well focused and it corresponds to the bottom wall of the microchannel. At the end of perfusion (t = 300 s), a sequence of *N* fluorescent images along the vertical *z*-dimension, previously defined *z*-stack, is acquired with a *z*-step of Δ*z* = 0.5 *μ*m. The first plane acquired is the focal plane identified as the adhering base plane during the perfusion, while the last one detected is represented by the last plane where the aggregates tips are visible. As a consequence, each *z*-stack has a proper height (*H*_*z*−*stack*_ < *h* = 100 *μ*m), defined as

$$\begin{array}{c} H_{z-stack}=\left(N-1\right)\;\Delta z. \end{array}$$ (2)

The impedance measurements are performed using the high precision LCR meter in the frequency range [1, 300] kHz with eight logarithmic spaced steps and a two-wires configuration. A drive voltage of 100 mV is chosen to avoid the redox reactions between electrodes and salts dissolved in plasma, since it is by far lower than the standard half-cell potential for gold (1.5 V). In particular, the impedance data used for the thrombus volume reconstruction are extracted from the impedance magnitude measured at 150 kHz, because, at this frequency, the lipidic membrane of platelets behaves like insulators and platelets aggregation can be measured by evaluating the impedance increase. For this reason, at 150 kHz the impedance temporal variations result more distinct and evident, thus facilitating the signal processing and interpretation. A total number of 31 experiments were conducted. All of them were analyzed to validate the *real-time* performances of our biosensor during the perfusion, but only for 22 of them thrombus volume was quantified from *z*-stacks, at t = 300 s. We did not analyze, in terms of final volume, the remaining 9 experiments because in those cases the channel was empty or completely obstructed by the thrombi, thus altering the microfluidic conditions, as shown subsequently.

### Volume estimation using the confocal microscope

In order to have a reference measurement to evaluate the performances of the developed device, at the end of each experiment (t = 300 s) the thrombus volume is computed using the confocal *z*-stacks, as reported in literature [2, 25, 26]. In particular, for each *z*-stack, a value in the gray-scale [0, 255] is selected as threshold value (*Thr*) with the aim of separating all pixels in two groups: those that exceed in intensity the threshold value and those that do not exceed it. Defining *f* = 0.33 *μ*m/pixel (1 pixel = 0.33 *μ*m) as the conversion factor from *μ*m to pixel, this method can be defined as Optical Thresholding (OT) and it allows to extract the area A_*i*_ (*μ*m^2^) covered by the objects, for each of the *N* images in the *z*-stack as

$$\begin{array}{c} A_{i}=\sum_{j=1}^{512}\sum_{k=1}^{512}pixel_{jki(intensity\;\geq \;Thr)}\;f^{2}\;\;\;\;\;\;\;i=1,...N. \end{array}$$ (3)

The thrombus volume *V*_*OT*_ (*μ*m^3^) related to the *z*-stack is quantified as

$$\begin{array}{c} V_{OT}=\Delta z\;\sum_{i=1}^{N}\left(A_{i}\right). \end{array}$$ (4)

**Fig 3:**
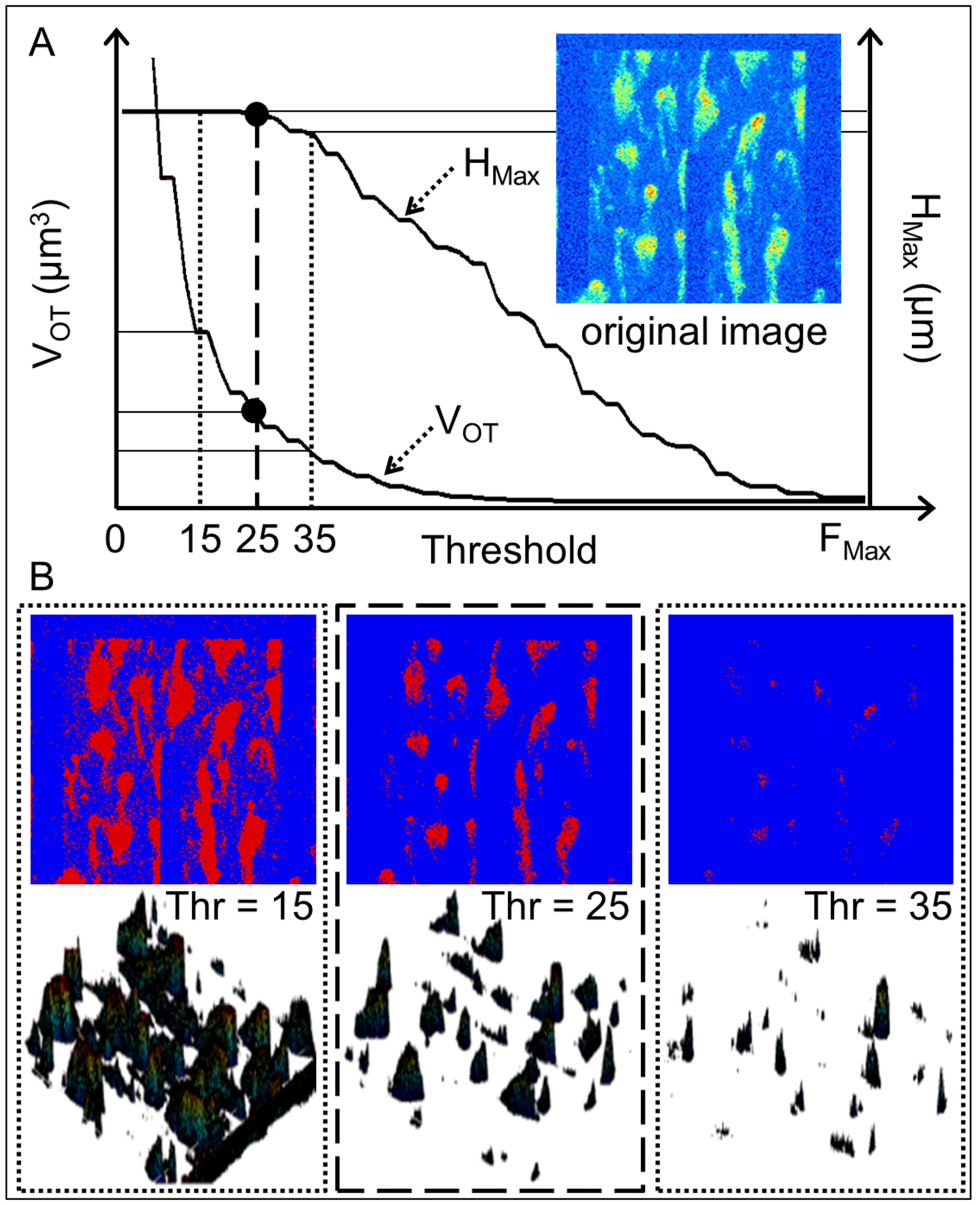
Automatic thresholding for thrombus volume quantification with confocal microscope. A: The optimal threshold is at the elbow point of the volume curve, corresponding to the point of saturation in the height curve (example value: 25). B: Binary mask representation and related 3D volume reconstruction, obtained with absolute threshold 15, 25 and 35, respectively (example values).

With the purpose of compensating the subjectivity in choosing a threshold value, here we propose a novel, fast and accurate thresholding method, an example of which is shown in Fig 3. The whole set of the gray-scale values [0, 255] is considered. With the thresholding method, for each *z*-stack acquired at t = 300 s, the volume *V*_*OT*_ and the maximum height *H*_*Max*_ reached by the thrombi are considered as functions of the threshold, varying from 0 to the maximum fluorescence value in the *z*-stack, *F*_*Max*_, as shown in Fig 3A. Observing the curves relative to *V*_*OT*_ and *H*_*Max*_ it is evident that, lowering the value of the threshold integer starting from *F*_*Max*_, *H*_*Max*_ gradually saturates to the value of *H*_*z*−*stack*_ and, correspondingly, *V*_*OT*_ diverges towards increasingly higher values (Fig 3A). This corresponds to a movement from a situation of underestimation (small amount of low thrombi) to an overestimation state (overly huge thrombi) of thrombus volume. The optimal threshold value (i.e. 25 in Fig 3A) is identified and chosen as the point of saturation of the height curve, corresponding to the elbow point of the volume curve. The visual comparison between one of the original images, shown in Fig 3A, and the related area A extracted with three different threshold values (i.e. 15, 25 and 35, Fig 3B) represents a graphical example of what just described. On one hand, lowering the threshold value with respect to the optimal value (i.e. 15 in Fig 3), the area A extracted leads to reconstruct artifacts caused by the optical noise and thrombi appear of square shape instead of pyramidal (Fig 3B, left panel). On the other hand, thresholds higher than the optimal one (i.e. 35 in Fig 3, right panel) lead to an underestimation of the real thrombus height and volume. As shown, volume quantification and reconstruction is dramatically different if the threshold value differs, in terms of absolute integer in the range [0, 255], of ± 10 from the optimal one (Fig 3B, middle panel).

#### Uncertainty evaluation

**Fig 4:**
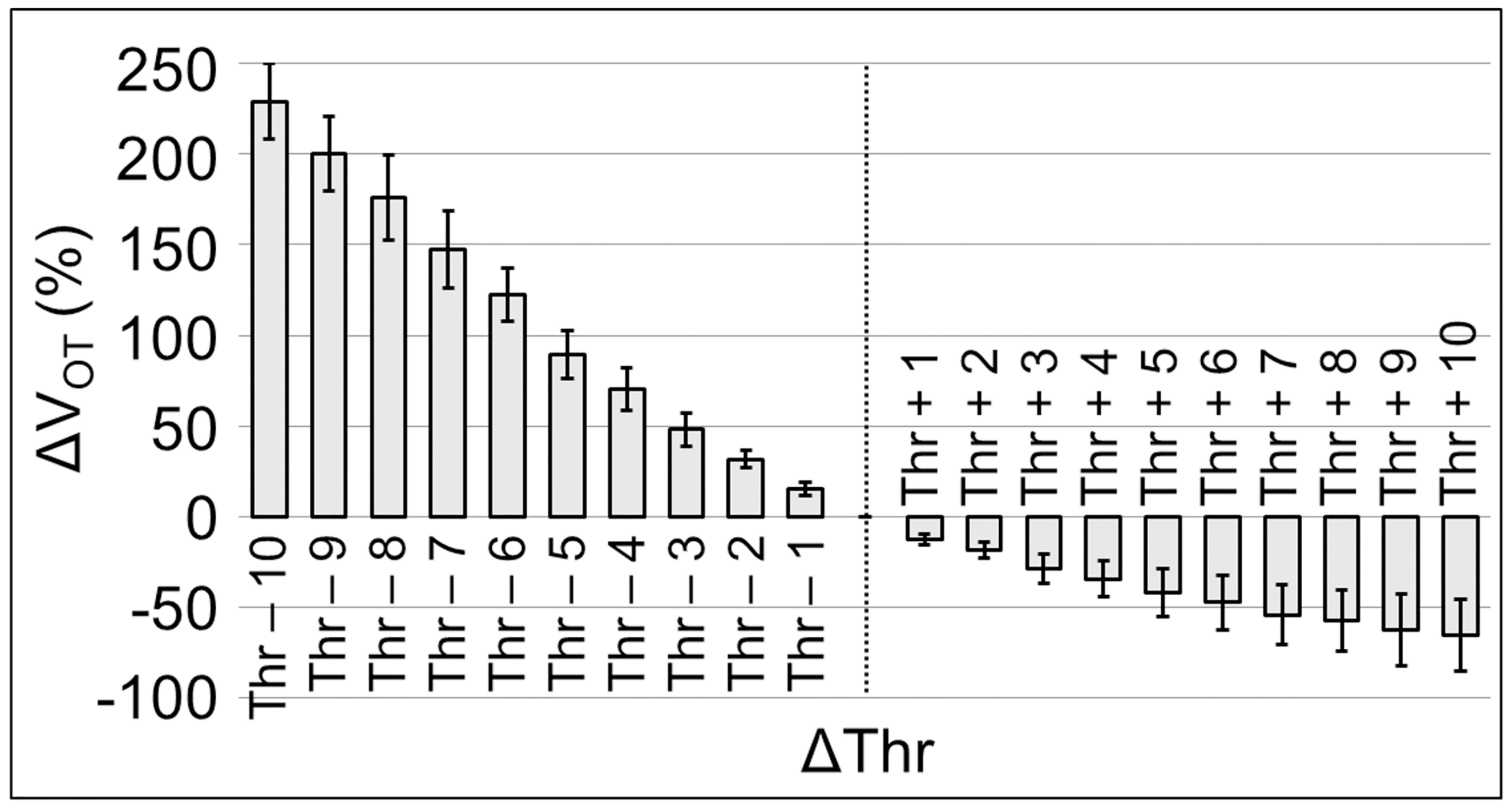
Sensitivity analysis of the threshold method. Volumes (n = 22) are expressed as percentage variation with respect to the volume obtained with the optimal threshold *Thr* (dashed line).

Remembering that an accurate quantification of thrombus volume with the optical thresholding represents a fundamental reference measurement to validate our impedance biosensor performances, the uncertainty *u*(*V*_*OT*_) in the computation of *V*_*OT*_ is calculated, from the uncertainties propagation theory [27], as

$$\begin{array}{c} u\left(V_{OT}\right)=\left(\frac{\partial V_{OT}}{\partial Thr}\right)\;u\left(Thr\right), \end{array}$$ (5)

where *Thr* is the threshold defined from the curves of Fig 3, as described, and *u*(*Thr*) is its related uncertainty. Looking at Fig 3A, it is possible to see that the *H*_*Max*_ and the *V*_*OT*_ curves have a stepwise behavior and the maximum step size for both is 3 threshold units; this step length corresponds, in the worst case, to an error in threshold identification of *δThr* = 3. Assuming an uniform distribution, the uncertainty in the optimal threshold value definition can be reasonably expressed as $u\left(Thr\right)=\delta Thr/\sqrt{3}=3/\sqrt{3}$. Thus, using the Eq (5), *u*(*V*_*OT*_) results in the order of 15%. Fig 4 represents the results of the sensitivity analysis of the optical thresholding method. For 22 *z*-stack, *V*_*OT*_ was evaluated for different threshold values in the range [*Thr* − 10, *Thr* + 10], where *Thr* is the optimal value identified as threshold for a specific *z*-stack (i.e. 25 in Fig 3). Therefore, the variation of *V*_*OT*_ as a function of the threshold was expressed as percentage variation of the volume quantity Δ*V*_*OT*_ (%) with respect to the related volume obtained with the optimal threshold value *Thr*

$$\begin{array}{c} \Delta V_{OT(Thr+i)}=100\;\frac{V_{OT(Thr+i)}-V_{OT(Thr)}}{V_{OT(Thr)}}\;\;\;\;\;i=-10,...10. \end{array}$$ (6)

The analysis results shown in Fig 4 (where Δ*V*_*OT*(*Thr*−1)_ = 15.38 ± 3.67% and Δ*V*_*OT*(*Thr*+1)_ = −12.41 ± 2.61%) are perfectly in agreement with the uncertainty of 15% obtained from the Eq (5). In general, it is remarkable to notice that the volume estimation using confocal microscope is highly sensitive to the intensity threshold variation; an error *δThr* of few threshold units can lead to an error in volume reconstruction greater than 100%. The method just described allows to establish a threshold value, not delegating that action to the human subjectivity of the operator and increasing the accuracy in thrombus volume quantification and reconstruction. The results in the text are expressed as mean ± standard deviation.

### Volume estimation using the new developed device

Electrical impedance data and 2D optical images are acquired simultaneously, as stated; the geometry of the thrombi is reconstructed from a solid model deduced from the 2D optical image of pixel fluorescence intensity. The thrombus height is assumed to be linearly proportional to the emission intensity of labeled platelets, as well established in literature [28]. Thus, pixel intensity is proportional to the thrombus height with respect to the microchannel surface, with a unique scale factor for all the pixels. The 3D solid model is derived in FUSEIT from fluorescence intensity values and the measured impedance; in FUSEIT the geometry is iteratively simulated changing the height scale factor until the simulated impedance matches the measured one; the thrombus volume from Impedance Measurements (*V*_*IM*_) is then calculated as previously described in detail in [19, 23].

## Results and discussion

A total number of 31 perfusion experiments (S1 Dataset) were performed for a duration of 300 seconds. During each experiment, the CCD camera acquired 2D images at 1 frame per second, for a total number of 300 images and, simultaneously, the new device acquired impedance data. At the end of perfusion (t = 300 s) a *z*-stack was acquired in order to evaluate the accuracy of the volume estimation provided by the new device here presented. In this Section, the new device performances are shown, starting from thrombus parameters evaluation, passing to the *real-time* identification of unstable thrombi, and finally showing the comparison between the volume estimated with Optical Thresholding and from Impedance Measurements (*V*_*OT*_ and *V*_*IM*_, respectively), with their 3D geometrical reconstruction.

### Thrombus geometrical and electrical parameters estimation

Accordingly to the acquisition criteria of confocal sections along *z*-dimension, the maximum height reached by the thrombi results H_*z*−*stack*_ = 43 ± 7 *μ*m (n = 31). Blood conductivity is estimated as *σ*_*blood*_ = 0.59 ± 0.19 S/m, at a measured hematocrit percentage of 42.60 ± 1.98%, values that appear perfectly in agreement with previously published data [29, 30]. This quantity is estimated by FUSEIT, starting from the impedance value at the beginning of each experiment, when no thrombus is formed. The thrombus conductivity should be, in this frequency range, ideally zero. However, thrombus is not a full solid geometry but it is an agglomerated structure of platelets with many interstitial spaces filled with plasma, that is conductive. From this observation, we started an experimental analysis, assigning a value to thrombus conductivity as a fraction of blood conductivity. The best results in terms of fit between *V*_*OT*_ and *V*_*IM*_ were obtained by setting the thrombus conductivity in the order of one eighth with respect to blood conductivity; the thrombus conductivity thus results to be *σ*_*thrombus*_ = 0.07 ± 0.02 S/m.

### *Real-time* monitoring of thrombus formation

**Fig 5:**
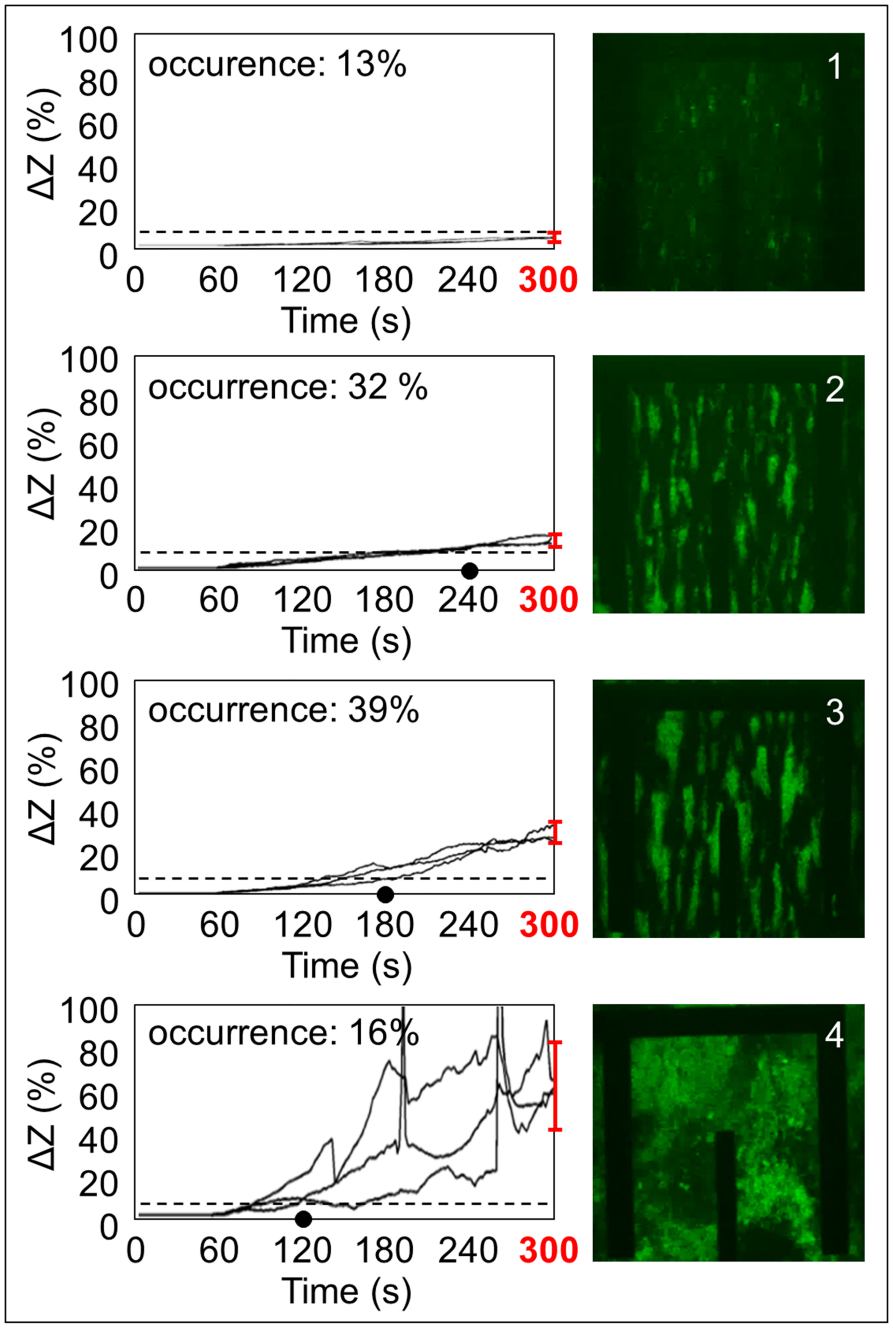
Classification of blood behavior. Classification of blood behavior in terms of platelet adhesion and aggregation, according to the variation of the impedance signal in time, expressed as relative increase over the initial value (Δ*Z* (%)). For each group, three representative signals of impedance variation (acquired in *real-time* during the perfusion) and a 2D image (acquired at t = 300 s) are shown as characteristic of the group. The red bars at t = 300 s show the percentage level of Δ*Z* overcome by all the signals of the related group within the end of perfusion (group 1: 7.55 ± 1.42%; group 2: 16.55 ± 1.88%; group 3: 31.15 ± 5.43%; group 4: 65.46 ± 20.12%). The dot on the time axis indicates the instant time at which all the signals of the group pass the 10% (dashed line) of impedance increase (group 1: signals do not overpass the 10% threshold within 300 seconds; group 2: dot at t = 240 s; group 3: dot at t = 180 s; group 4: dot at t = 120 s).

**Fig 6:**
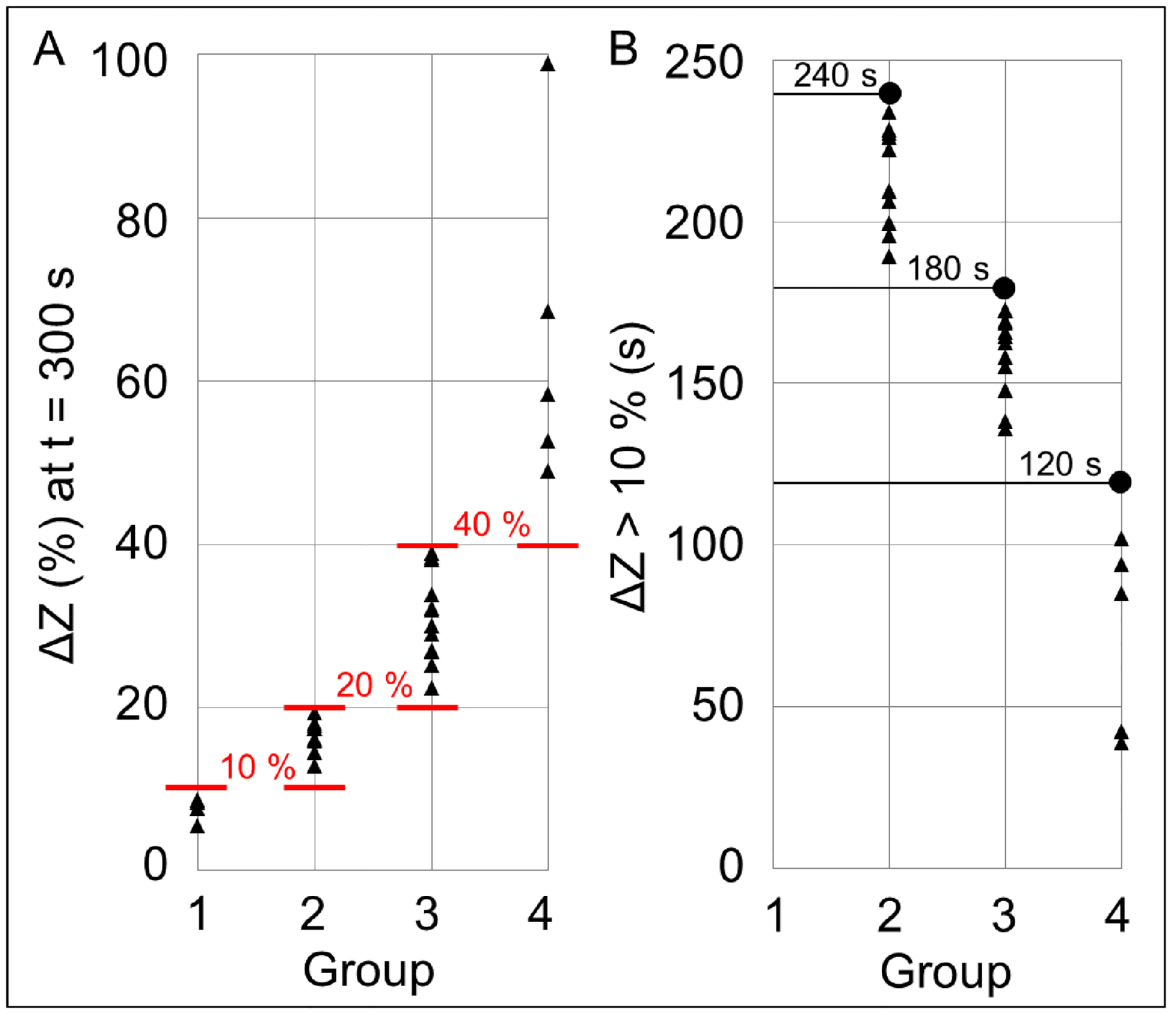
Extraction of classification thresholds. The overall increase of the impedance magnitude within 300 seconds and the intermediate time instant of 10% threshold overpassing are experimentally defined, for each group, from the corresponding values of each experiment. Group 1: Δ*Z* = 7.55 ± 1.42% (always < 10%). Group 2: Δ*Z* = 16.55 ± 1.88% (10% < Δ*Z* < 20%); Δ*Z* > 10% at t = 213.83 ± 15.76 s (dot at t = 240 s). Group 3: Δ*Z* = 31.15 ± 5.43% (20% < Δ*Z* < 40%); Δ*Z* > 10% at t = 159.32 ± 12.63 s (dot at t = 180 s). Group 4: Δ*Z* = 65.46 ± 20.12% (Δ*Z* > 40%); Δ*Z* > 10% at t = 72.37 ± 29.81 s (dot at t = 120 s).

The non-invasive and *real-time* analysis of the impedance magnitude measured at 150 kHz permits to monitor adhesion and aggregation processes not only at the end of each experiment from *z*-stacks, but also during the entire perfusion time. This new way of monitoring in *real-time* the blood behavior under dynamic conditions makes concretely possible to identify experimental parameters useful for a fast evaluation and prediction of the adhesion and aggregation levels that will be reached during the whole perfusion time (in particular, at t = 300 s), as shown in Fig 5. Considering and representing the relative variation of impedance magnitude with respect to its initial value (Δ*Z* (%)), it is possible to distinguish four different physiological blood behaviors in terms of platelet adhesion and aggregation, among the 31 experiments performed. In Fig 5, each classification group is illustrated by three representative signals of impedance variation in time during the perfusion (from t = 0 s to t = 300 s) and, on the right, by a representative 2D image. The single image is acquired at t = 300 s and is related to the plane of electrodes, where platelets adhere. Observing Fig 5, the red bars at t = 300 s show the percentage level of Δ*Z* overcome by all the signals of the related group within the end of perfusion. The dot on the time axis indicates the instant time at which all the signals of the group pass the 10% of impedance increase (dashed line). The overall increase of the impedance magnitude within 300 seconds and the intermediate time instant of 10% threshold overpassing are experimentally defined, for each group, from the corresponding values of each experiment, whose distribution is illustrated in Fig 6. These values allow a classification of blood behavior and, in addition, they turn out to be effectively useful to predict in *real-time* the levels of adhesion and aggregation that will be reached at the end of perfusion, as confirmed by the 2D representative images shown in Fig 5. Group 1 (occurrence: 13% of experiments, 4 of 31) is characterized by very low adhesion without evident aggregation, with the relative impedance increase Δ*Z* that does not reach the 10% (7.55 ± 1.42%), not even after 300 seconds of blood perfusion (Figs 5 and 6). Group 2 (occurrence: 32% of experiments, 10 of 31) is characterized by regular adhesion and aggregation, where Δ*Z* exceeds the 10% increase within 240 s (213.83 ± 15.76 s) and reaches an increase grater than 10% (16.55 ± 1.88%) at t = 300 s (Figs 5 and 6). Group 3 (occurrence: 39% of experiments, 12 of 31) is characterized by strong adhesion and aggregation; in this case, Δ*Z* exceeds the 10% increase within 180 s (159.32 ± 12.63 s) and reaches an increase between 20% and 40% (31.15 ± 5.43%) at t = 300 s (Figs 5 and 6). Group 4 (occurrence: 16% of experiments, 5 of 31) is characterized by very high adhesion with massive towering aggregation that leads to the microchannel occlusion; in this case Δ*Z* overcome 10% within 120 s (72.37 ± 29.81 s) and the 40% (65.46 ± 20.12%) at t = 300 s (Figs 5 and 6).

**Fig 7:**
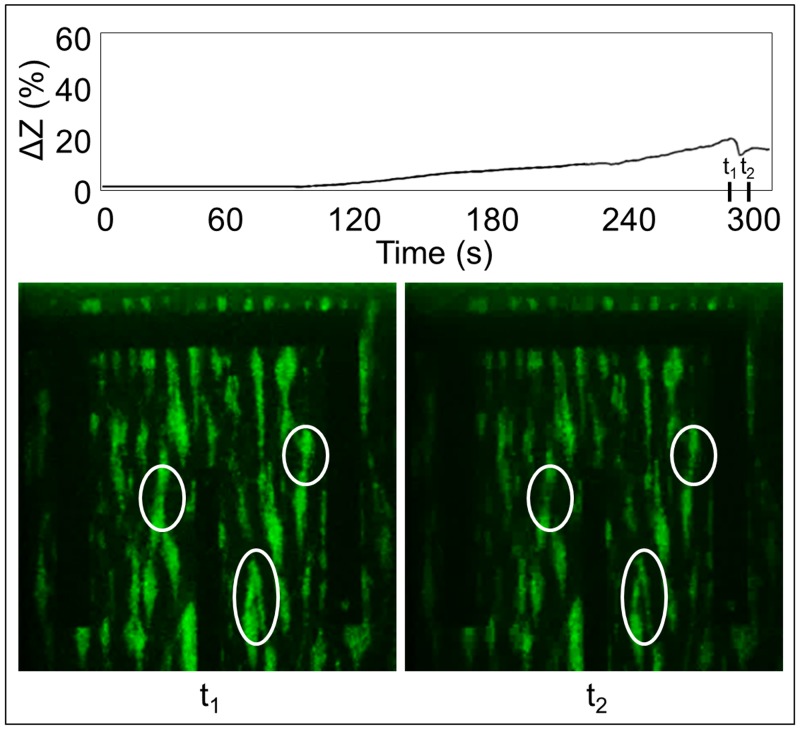
*Real-time* monitoring and identification of critical events. *Real-time* identification of weakening and detaching aggregates and morphologic variations of their distribution based on the impedance increase (Δ*Z* (%)) evaluation in time. Images at time instant t = t_1_ (286 s) and t = t_2_ (292 s) show the distribution of the aggregates before and after the event, respectively. Circles underline the greater variations.

The novelty of this new methodology is also represented by the possibility to identify and follow in *real-time* instantaneous and rapid events of detachment (quantity deviation) of the aggregates or morphologic variation (quality deviation) in their spatial distribution. In fact, in the attempt to monitor and control the thrombotic risk and analyze in *real-time* the dynamic of thrombus formation events, Fig 7 shows the ability of identifying instantaneously the structural variation and the detachment of aggregates. In particular, only observing the time trend of the impedance signal Δ*Z* (%) in Fig 7, it is possible to identify and quantify the sudden and rapid signal falling that occurs between the time instants t_1_ (i.e. 286 s in Fig 7) and t_2_ (i.e. 292 s in Fig 7). Images in Fig 7 show, and visibly demonstrate, the variations in terms of aggregate detachment and morphologic variations of volume distribution (particularly evident in the circles), before and after the signal falling (at t = t_1_ and t = t_2_, respectively).

### Reconstruction of 3D volume distribution with the new device

**Fig 8:**
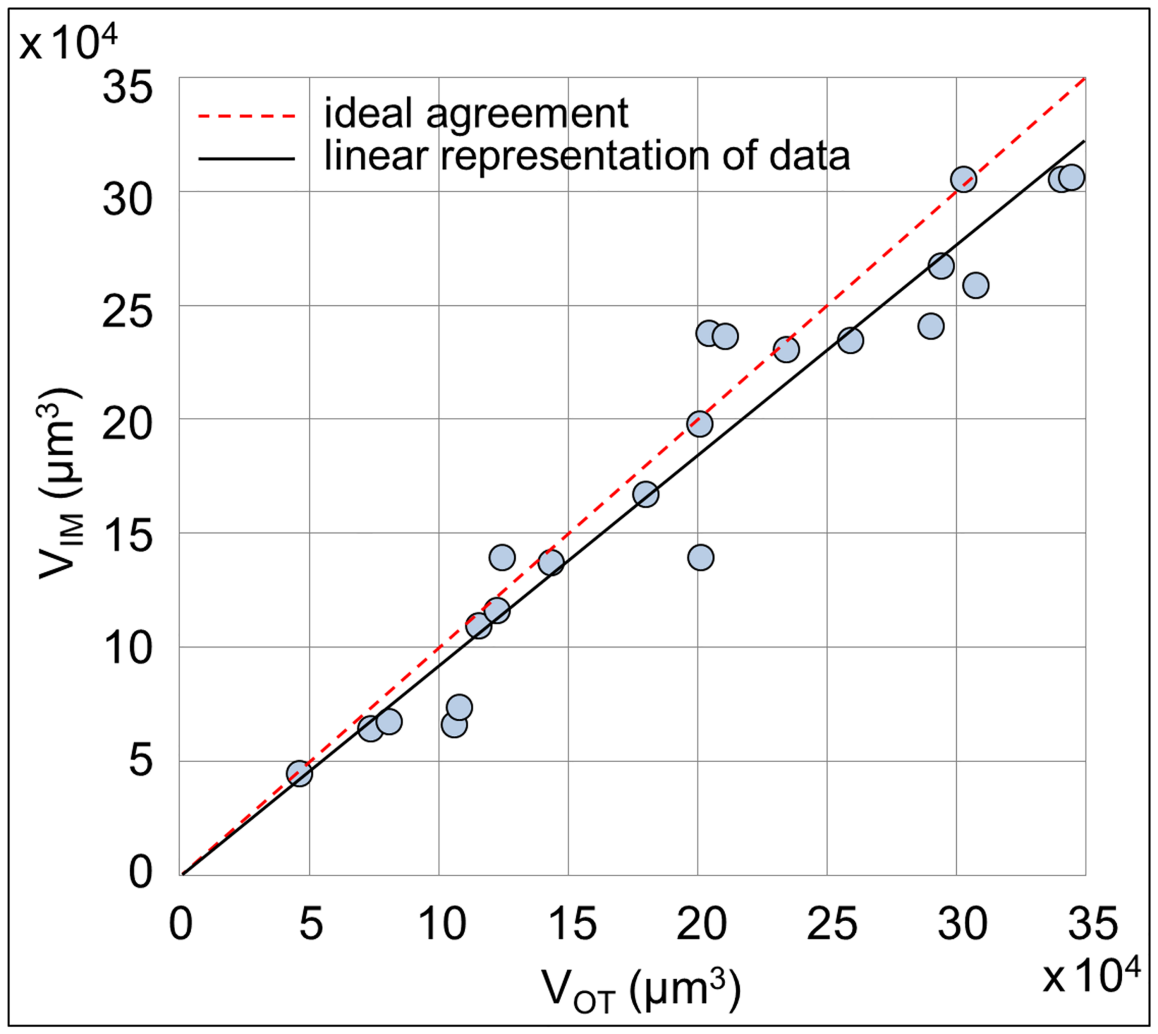
Volumes comparison. Comparison between volumes *V*_*OT*_ and *V*_*IM*_ obtained at t = 300 s from optical and impedance data (n = 22). Data are highly correlated and exhibit a Pearson’s correlation coefficient *r* equal to 0.96 (*p* value < 0.01). The linear representation of data (black line) is shown together with the ideal agreement condition (red dashed line).

**Fig 9:**
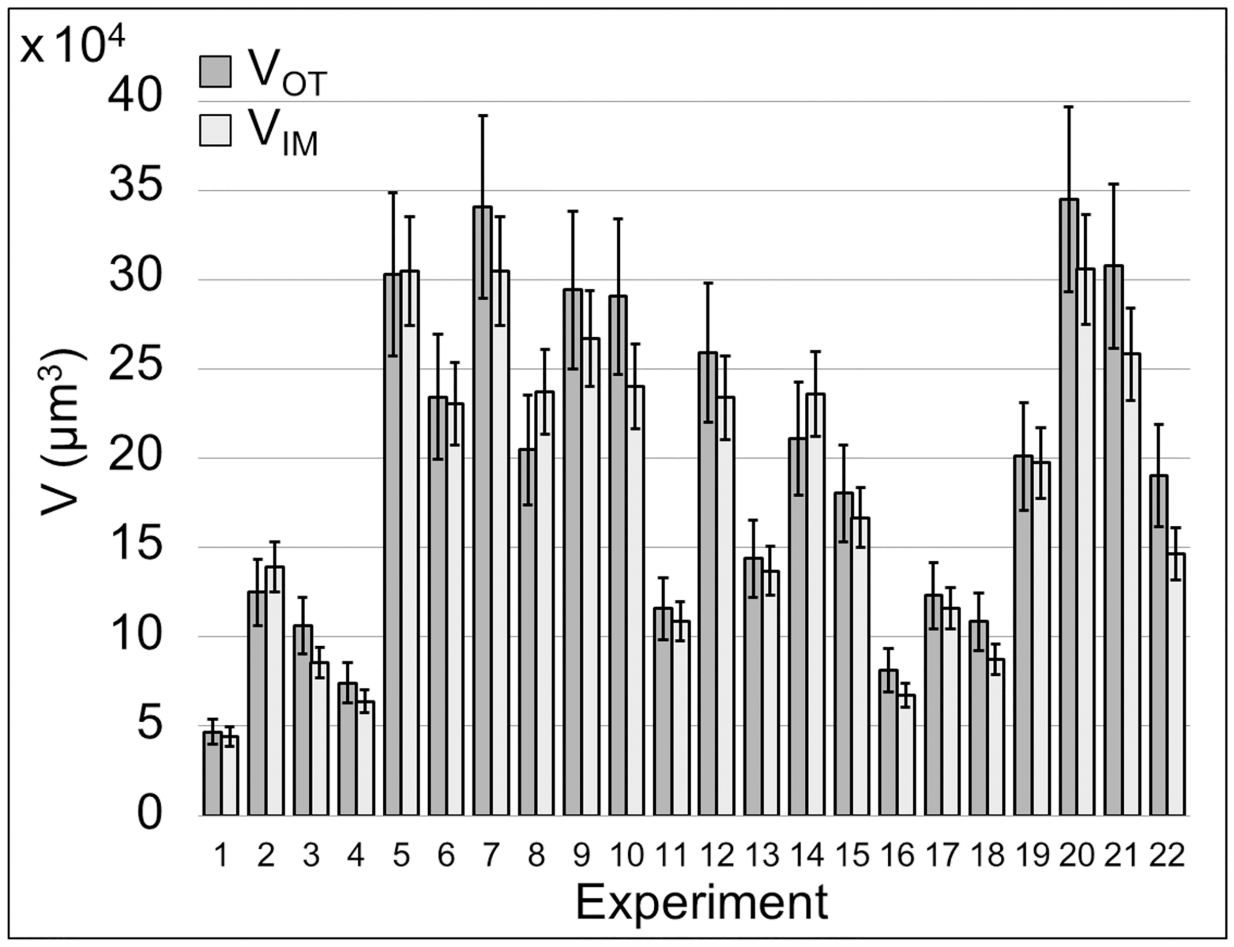
Compatibility between *V*_*OT*_ and *V*_*IM*_. For each experiment (n = 22), *V*_*OT*_ is expressed as the specific *z*-stack value ±15% (uncertainty of thresholding method), while *V*_*IM*_ is expressed as the specific *z*-stack value ±10% (uncertainty of reconstruction from impedance data). *V*_*OT*_ and *V*_*IM*_ measurements appear compatible and, for our scopes, interchangeable.

**Fig 10:**
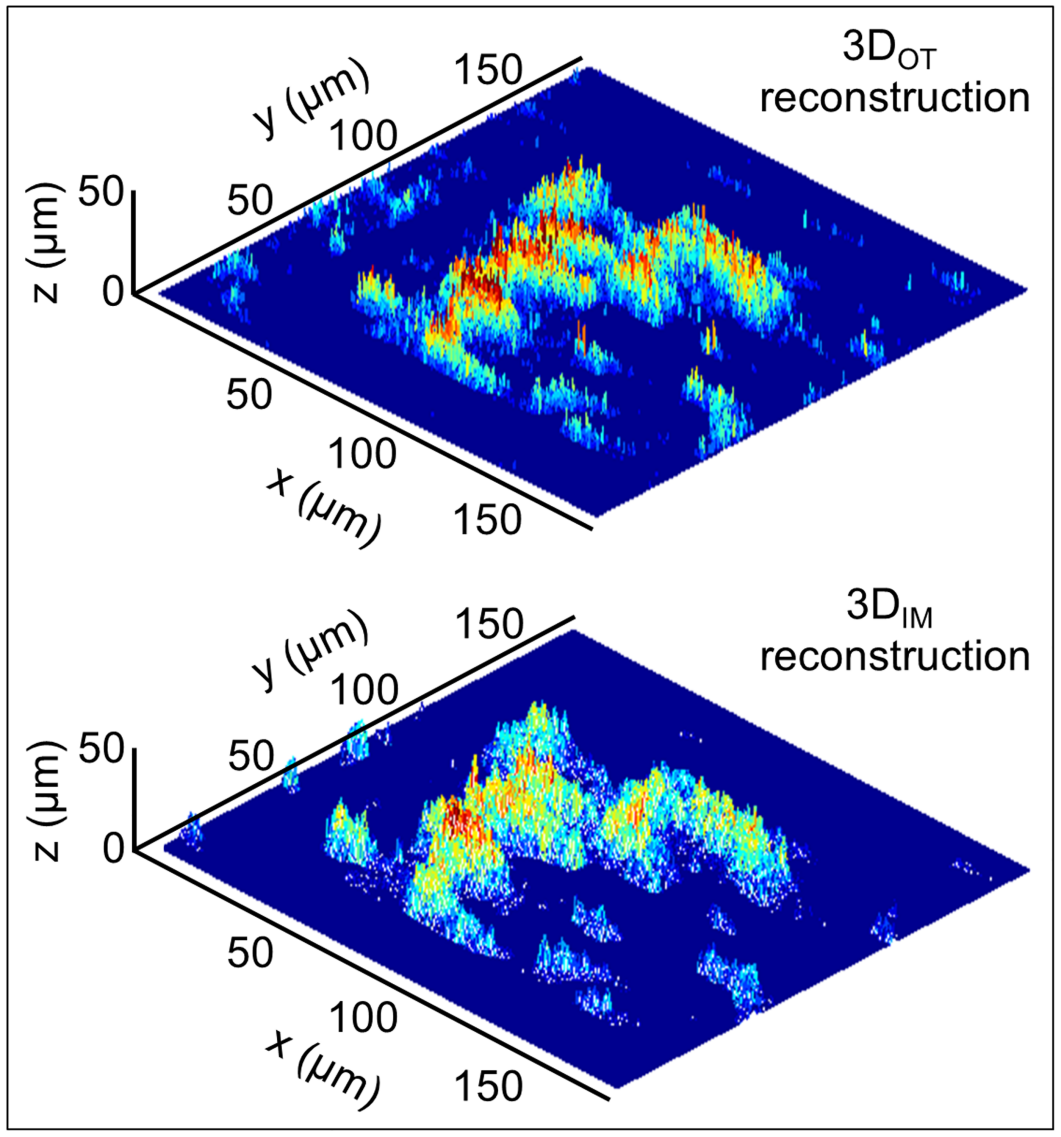
Three-dimensional rendering. Example of a 3D representation of volume distribution obtained with Optical Thresholding (3D_*OT*_ reconstruction) and with Impedance Measurements (3D_*IM*_ reconstruction) at t = 300 s.

The comparison between *V*_*OT*_ and *V*_*IM*_, shown in Fig 8, reveals that they are highly correlated to each other exhibiting a Pearson’s correlation coefficient [31]*r* equal to 0.96 (*p* value < 0.01). Data are obtained, as previously explained, from 22 experiments (71% of total), corresponding to experiments classified as group 2 and 3, as shown in Fig 5. In detail, only the experiments associated to a physiological (groups 2-3) and not pathological (groups 1-4) blood behavior were used to compute *V*_*IM*_ and comparing them to the related *V*_*OT*_ with the aim of validating the impedimetric method. This not because of detection limits of the new biosensor but because the traditional optical thresholding method shows limits about the accuracy in quantifying excessively small or huge volumes. Despite this distinction, the experiments classified as group 1 and 4 represent extreme blood behavior conditions that our biosensor is able to recognize in *real-time* and classify, correctly and accurately, as pathological situations, as previously described. Fig 9 presents, for each *z*-stack of groups 2 and 3, *V*_*OT*_ and *V*_*IM*_ with their uncertainty. According to what previously described, *V*_*OT*_ are expressed with 15% uncertainty, while *V*_*IM*_ with 10% uncertainty, as previously published in [23]. The two methodologies for the volume measurement are metrologically compatible. This confirms the accuracy of our new device measurements, whose methodology is based on fusion of 2D fluorescent images with electrical impedance data. The use of the confocal microscope has proved, at this stage, to be necessary to compare and validate the performances of our biosensor. In Fig 10 two representative 3D reconstructions of the volumes spatial distribution are shown; the upper one is obtained from Optical Thresholding method (3D_*OT*_ reconstruction), the lower one by FUSEIT from Impedance Measurements (3D_*IM*_ reconstruction). The two methods provide very close results.

## Conclusions

Medical research has moved gradually its attention to new non-invasive and *real-time* devices able to assess the thrombotic risk profile by reconstructing the dynamic of thrombus formation events and thus allowing the monitoring of anticoagulant and antiplatelet therapies. In the specific biological field of blood coagulation and its related diseases, the accuracy and the standardization of thrombus volume measurements, starting from confocal microscope images, have always been subject of intense investigation. However, the translation in clinical practice has been prevented because of the high costs and dimensions. Impedance measurements applied to blood analysis have received a considerable attention because of *real-time* monitoring of the *in vitro* thrombus formation.

In order to improve the thrombus measurement accuracy, we performed perfusion experiments for a total time of 300 seconds, measuring impedance magnitude at 150 kHz and acquiring, simultaneously, optical images. At the end of each blood perfusion (t = 300 s), we acquired a *z*-stack which elaboration allowed us to confirm the validity of the new device performances. After comparing the results obtained with the two methods and verifying their interchangeability, we can say that the *real-time* monitoring of the individual hemostatic behavior is possible only through the acquisition of the impedance signals and their representation in terms of relative variation of impedance magnitude Δ*Z* (%).

In this paper, we described a fast, competitive and innovative device with a method for the *in vitro* determination of thrombus formation under flow. In particular, it is capable of discriminating different hemostatic conditions as demonstrated by the presented preliminary results. Our method should be helpful to evaluate *in vitro* the thrombotic risk of individuals affected by cardiovascular diseases or other thromboembolic disorders. However, large clinical studies should be undertaken to investigate the impact of our *in vitro* methodology and thus contribute to a more accurate understanding of thrombosis and antiplatelet therapy resistance. According to the obtained results, our biosensor can be proposed as a Point of Care (POC) device to adequately monitor antiplatelet therapies and for the construction of models for optimizing anticoagulant treatment.

## Supporting information

S1 Dataset Raw data obtained from all the experiments performed. (XLSX)
